# The Impact of Reference Standard on Diagnostic Testing Characteristics for Carpal Tunnel Syndrome: A Systematic Review

**DOI:** 10.1097/GOX.0000000000005067

**Published:** 2023-07-03

**Authors:** Olivia M. Bennett, Erika D. Sears

**Affiliations:** From the * University of Michigan Medical School, Ann Arbor, Mich.; † Department of Surgery, Section of Plastic Surgery, Michigan Medicine, Ann Arbor, Mich.; ‡ Veterans Affairs Center for Clinical Management Research, VA Ann Arbor Healthcare System, Ann Arbor, Mich.

## Abstract

Lack of a reliable reference standard for carpal tunnel syndrome (CTS) diagnosis could impact the diagnostic test characteristics. This systematic review sought to evaluate differences in the accuracy of CTS diagnostic modalities based on the reference standard used.

**Methods:**

A systematic review was performed following PRISMA guidelines to investigate diagnostic modalities used in CTS. A literature search of Embase, PubMed, and Cochrane Reviews was conducted for the years of 2010–2021 for primary data, and 113 studies met final inclusion criteria. Studies were stratified based on the reference standard utilized and diagnostic modality assessed, and the weighted means of the sensitivities and specificities were calculated.

**Results:**

Thirty-five studies used clinical diagnosis alone as a reference standard, and 78 studies used electrodiagnostic study (EDS). The specificity for MRI and ultrasound (US) were substantially lower when EDS was used as the reference standard. MRI was the test most affected by the reference standard used, showing increased sensitivity when using EDS as the reference compared to clinical diagnosis (77.1% versus 60.9%) and decreased specificity (87.6% versus 99.2%). Regardless of the reference standard used, all tests had anticipated false-positive and/or false-negative rates of at least 10%.

**Conclusions:**

Testing characteristics vary greatly based on the choice of reference standard, with the sensitivity of MRI most affected. Regardless of reference standard used, EDS, US, and MRI each had false-positive and/or false-negative rates too great to be appropriate for use as a screening examination.

**Takeaways**

**Question:** How accurate are diagnostic tests in the diagnosis of carpal tunnel syndrome and how does the chosen reference standard impact these findings?

**Findings:** We performed a systematic review of primary data investigating electrodiagnostic studies, ultrasound, and MRI to determine the accuracy of these tests in CTS diagnosis when different reference standards were used. All tests had greater than 10% false-positive or false-negative rates regardless of the reference standard used.

**Meaning:** Neither MRI, US, nor EDS are adequately sensitive or specific to be used as screening modalities for CTS.

## INTRODUCTION

Carpal tunnel syndrome (CTS) affects approximately 3%–6% of adults and is the most common compression neuropathy in the general population.^1,2^ Although CTS is a clinical diagnosis, no gold standard exists for definitive diagnosis. Electrodiagnostic study (EDS) is often used routinely by general practitioners and surgeons, along with other diagnostic modalities, for diagnostic support when CTS is clinically suspected.^3^ However, research assessing performance characteristics of diagnostic tests for CTS is limited by the lack of a consistent reference standard.

The unique benefits of the different testing modalities used in CTS diagnosis have been widely debated.^8–10^ In 2016, the American Academy of Orthopedic Surgeons released an Evidence-Based Clinical Practice Guideline on Management of Carpal Tunnel Syndrome in which recommendations were made for or against the routine use of various diagnostic tools, such as EDS, MRI, ultrasound (US), and diagnostic scales.^4^ Although these recommendations were based on available literature through 2015, it remains unclear how test performance characteristics vary based on differences in the reference standard used, which may include clinical diagnosis, EDS, or symptomatic improvement after surgical treatment.

Our study sought to evaluate variation in the use of reference standards among CTS diagnostic tests and the impact on testing characteristics based on the reference standard used. Specifically, we evaluated testing characteristics (ie, sensitivity and specificity) based on the two most utilized reference standards—clinical diagnosis alone and EDS with or without the support of clinical diagnosis. By evaluating the diagnostic modalities in this manner, we can consistently compare diagnostic test performance characteristics. We hypothesize that sensitivity and specificity values measured using clinical diagnosis as the reference standard will differ from performance characteristics measured using EDS as the reference standard.

## METHODS

### Search Strategy

The study was performed according to the Preferred Reporting Items for Systematic Review and Meta-Analysis guidelines. Cochrane Review, PubMed, and Embase were searched for the literature from 2010, to May 26, 2021, using “carpal tunnel syndrome” and “diagnosis” as the key word search in different combinations. This timeline was selected to include relevant articles published after the American Academy of Orthopedic Surgeons’ most recent practice guideline, while also including the more recently published data in the last decade. Search terms are outlined in Table 1.

**Table 1.** Search Terms*

	
PubMed†	(carpal tunnel syndrome[mh] OR carpal tunnel[tiab]) AND (diagnosis[mh] OR diagnoses[tiab] OR diagnosis[tiab] OR diagnostic[tiab] OR diagnostics[tiab]) AND 2010: 2021[dp]
EMBASE‡	(“carpal tunnel syndrome”:ab,ti OR “carpal tunnel”:ab,ti) AND (“diagnoses”:ab,ti OR “diagnosis”:ab,ti OR ‘diagnostic’ab,ti OR ‘diagnostics’ab,ti) AND[2010-2021]/py
Cochrane§	(carpal tunnel syndrome):ti,ab,kw OR (carpal tunnel):ti,ab,kw AND (“diagnosis” OR “diagnoses” OR “diagnosis” OR “diagnostic” OR “diagnostics”):ti,ab,kw

* Publication date from January 2010 to May 2021; searches performed May 26, 2021.

† PubMed: mh = mesh heading, tiab = title and/or abstract, dp = date of publication.

‡ EMBASE: ab = abstract, ti = title, py = publication year.

§ Cochrane: ti = title, ab = abstract, kw = keyword. Specification added with Cochrane Library.

### Inclusion Criteria and Data

**Fig. 1.**
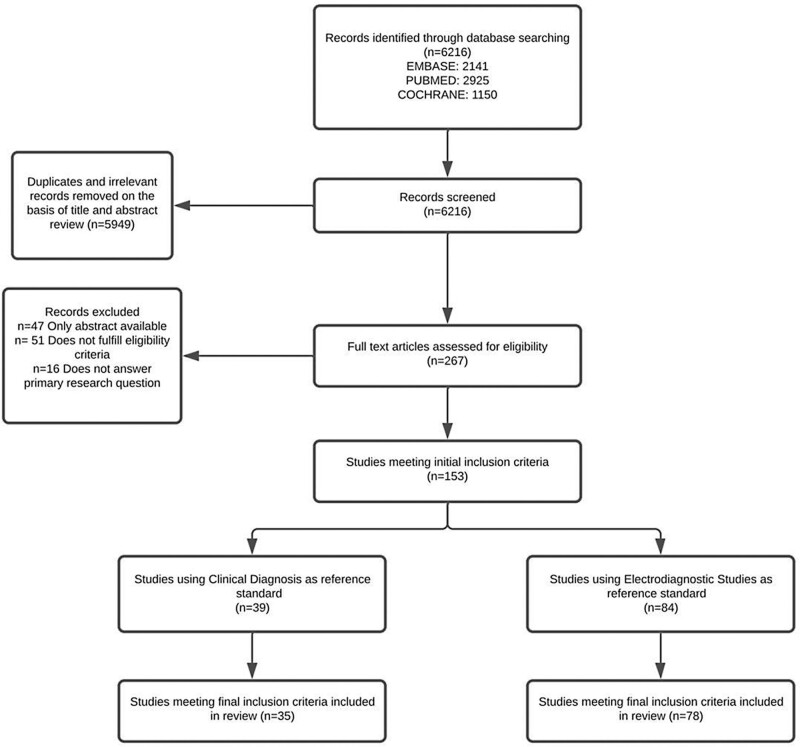
Flowchart of the selection of included articles.

Studies presenting primary data of testing characteristics of EDS, MRI, US, US elastography, clinical diagnosis, and diagnostic questionnaires were evaluated. Inclusion criteria included specifying a defined reference standard; including a minimum of 10 patients per diagnostic modality examined; presenting data on sensitivity, specificity, positive predictive value, negative predictive value, and/or area under the curve; and having a full-length article available in English. The inclusion criteria were broadly inclusive of published studies meeting the inclusion criteria to fully evaluate differences in the reference standard used and the impact on testing characteristics reported. Article screening and data abstraction were performed by a single author, which included extracting publication details as well as the number of patients evaluated, number of hands/wrists evaluated, reference standard used, and specific diagnostic tool(s) assessed. Specific values obtained by the study for sensitivity, specificity, positive predictive value, negative predictive value, and/or area under the curve were also recorded as available. For studies evaluating multiple different diagnostic cutoffs, anatomic landmarks, techniques, etc., testing characteristic values were taken for the method that gave the greatest specificity. Any areas of uncertainty in applying the inclusion criteria were discussed between the two authors until agreement was reached. A flowchart of studies evaluated is included in Figure 1.

Following data abstraction, studies using clinical diagnosis alone as the reference standard were isolated and evaluated by further inclusion criteria. Here, inclusion criteria included the use of clinical diagnosis without further diagnostic support as the reference standard; evaluation of EDS, MRI, and/or US (gray scale and/or power Doppler) performance; and presentation of sensitivity and/or specificity of the diagnostic tools evaluated. This process was repeated with studies using EDS as the reference standard, with further inclusion criteria including the use of EDS with or without the support of clinical diagnosis as the reference standard, evaluation of MRI and/or US (gray scale and/or power Doppler), and presentation of sensitivity and/or specificity.

### Statistical Evaluation

The mean sensitivity and specificity were calculated using a weighted average for each reference standard subgroup and diagnostic tool assessed. Weighted means were calculated using the number of hands/wrists included in the study. When the number of hands/wrists was not given, the number of patients was used for weighting purposes.

## RESULTS

The search strategy and initial title/abstract screen yielded a total of 267 full-text articles to assess for eligibility, with 153 articles found to meet the initial inclusion criteria (Fig. 1). Thirty-five studies used clinical diagnosis as the reference standard to evaluate EDS (26 studies, 4412 hands/wrists), MRI (three studies, 348 hands/wrists), and diagnostic US (20 studies, 3365 hands/wrists) performance. These included nine cross-sectional studies, eight cohort studies, and 18 case-control studies. Seventy-eight studies used EDS with or without clinical diagnosis as the reference standard to evaluate MRI (13 studies, 1246 hands/wrists) and US (68 studies, 10,187 hands/wrists). These included 38 cross-sectional studies, eight cohort studies, and 32 case-control studies. The diagnostic tests evaluated and their weighted mean sensitivity and specificity are outlined in Tables 2 and 3.^16-128^ Although studies utilizing “clinical diagnosis” as a reference standard were treated as a unified subset, many studies^16-128^ were found to utilize different definitions of clinical diagnosis. An outline of the utilized definitions for “clinical diagnosis” is presented in Supplemental Digital Content 1. (**See appendix, Supplemental Digital Content 1**, which shows the clinical diagnosis definitions in studies using clinical diagnosis as the reference standard, http://links.lww.com/PRSGO/C612.) Overall, in the period searched, US and EDS were evaluated more frequently than MRI. Among all of the testing modalities evaluated, all tests had at least 10% false-positive (EDS, US) or at least 10% false-negative rates (EDS, US, MRI) on average, regardless of the reference standard.

**Table 2.** Testing Characteristics for Studies Using Clinical Diagnosis as the Reference Standard

Diagnostic Test	Studies Included	Total Number of Hands/Wrists (n)	Mean Sensitivity, %*	Mean Specificity, %*
Electrodiagnostic tests	16, 17, 18, 19, 20, 21, 22, 23, 24, 25, 26, 27, 28, 29, 30, 31, 32, 33, 34, 35, 36, 37, 38, 39, 40, 41	4412	84.1	89.8
MRI	18, 42, 43	248	60.9	99.2
US	16, 18, 19, 20, 21, 23, 25, 28, 32, 33, 36, 37, 38, 39, 44, 45, 46, 47, 48, 49, 50	3,365	80.7	87.1

* Weighted by sample size.

US, ultrasound.

**Table 3.** Testing Characteristics for Studies Using Electrodiagnostic Studies with or without Clinical Diagnosis as the Reference Standard

Diagnostic Test	Studies Included	Total Number of Hands/Wrists (n)	Mean Sensitivity, %*	Mean Specificity, %*
MRI	51, 52, 53, 54, 55, 56, 57, 58, 59, 60, 61, 62, 63	1246	77.1	87.6
US	51, 55, 61, 64, 65, 66, 67, 68, 69, 70, 71, 72, 73, 74, 75, 76, 77, 78, 79, 80, 81, 82, 83, 84, 85, 86, 87, 88, 89, 90, 91, 92, 93, 94, 95, 96, 97, 98, 99, 100, 101, 102, 103, 104, 105, 106, 107, 108, 109, 110, 111, 112, 113, 114, 115, 116, 117, 118, 119, 120, 121, 122, 123, 124, 125, 126, 127, 128	10,187	76.2	84.8

* Weighted by sample size.

US, ultrasound.

When clinical diagnosis alone was utilized as a reference standard, MRI was found to be the most specific and least sensitive diagnostic tool evaluated with a specificity of 99.2% and a sensitivity of 60.9%. EDS was the most sensitive modality within this subset with a sensitivity of 84.1% but was the second most specific (89.8%) among MRI, EDS, and US. US was the least specific of the three diagnostic tools (87.1%) and was the second most sensitive testing modality (80.7%) when clinical examination was used as the reference standard.

More studies utilized EDS as a reference standard to evaluate MRI and US testing performance, with 78 studies using EDS as the reference standard compared to 22 studies that evaluated MRI and US using clinical diagnosis alone as the reference standard. When EDS with or without clinical diagnosis was used as the reference standard, MRI was more specific than US (specificity: 87.6% versus 84.8%, respectively), as well as more sensitive than US (sensitivity: 77.1% versus 76.2%, respectively).

**Fig. 2.**
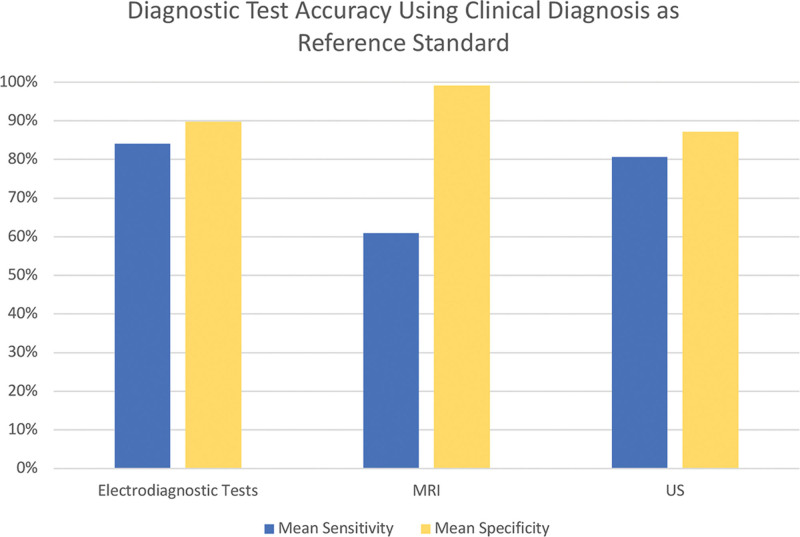
Mean sensitivity and specificity of various diagnostic modalities calculated from all included studies using clinical diagnosis as the reference standard. Reported means are weighted by sample size.

**Fig. 3.**
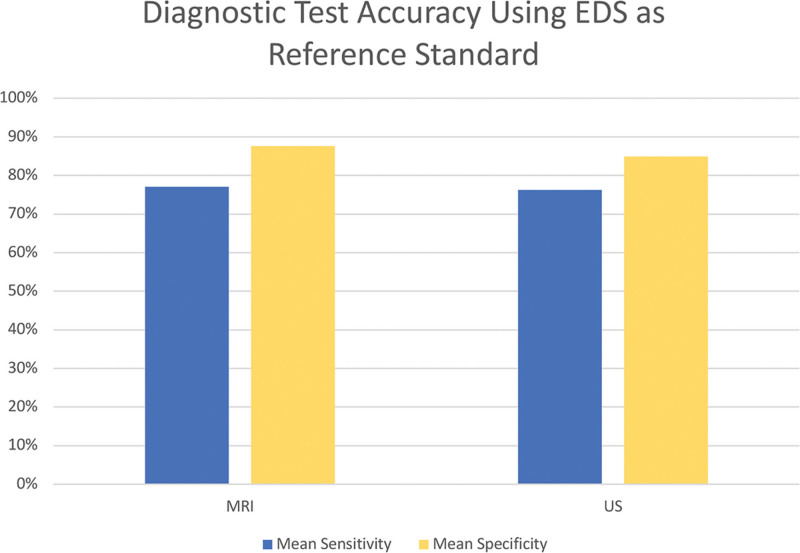
Mean sensitivity and specificity of various diagnostic modalities calculated from all included studies using EDS as the reference standard. Reported means are weighted by sample size.

Sensitivity and specificity of US as a diagnostic tool were greater when characteristics were assessed using clinical diagnosis as a reference standard compared to when EDS was used as the reference standard, with an increase in sensitivity of 4.5% and increase in specificity of 2.3%. A larger change was seen in the mean sensitivity and specificity of MRI based on differences in the reference standard, with an increase in specificity of 11.6% and a decrease in sensitivity of 16.2% when clinical diagnosis was the reference standard versus EDS (Figs. 2 and 3).

## DISCUSSION

This study found that diagnostic test performance for CTS differs based on the reference standard used. Although our results demonstrate that EDS had a relatively high sensitivity and specificity when clinical diagnosis was used as the reference standard, both imaging tests (MRI and US) saw a decrease in specificity when EDS was used as a reference compared to clinical diagnosis. Although EDS was more commonly utilized as the reference standard when assessing imaging the studies, MRI and US had a false-negative and/or false-positive rate of 10% or greater. EDS had the same limitation when clinical diagnosis was used as the reference with neither its sensitivity nor its specificity measuring above 90%.

Although no reference standard consensus exists for CTS diagnosis, nerve conduction studies are often utilized as a reference standard when assessing the validity of diagnostic tools. However, our study found a mean false-negative rate of 15.9% and a false-positive rate of 10.2% for EDS. Our findings are similar to previous studies which have reported false-negative rates ranging from 16% to 34%^5^ and false-positive rates ranging from 2.5% to 43% with variation due in part to different cutoff values used.^6,7^ These studies can be uncomfortable and expensive, and their high rate of false positives and false negatives support proponents of avoiding routine EDS use for all CTS patients. Similarly, MRI and US had false-negative rates and/or false-positive rates greater than 10% regardless of the reference standard used, suggesting that these studies should be avoided as screening examinations as well.

EDS is often used to assess the severity of CTS which may have value in determining the appropriate treatment course or counseling patients on anticipated outcomes. Studies have also suggested that ultrasonography may play a beneficial role in determining CTS severity,^8^ as the modality carries the added benefit of identifying structural abnormalities which may contribute to symptoms.^9^ MRI similarly has an added advantage of identifying structural abnormalities^10^ although its utility is limited by the expense and time involved in MRI evaluation. Measurements of dimensions of the median nerve and other structures within the carpal tunnel may help identify the extent of pathologic changes, and these modalities may therefore be useful when stratifying cases based on severity.

This study also found that the weighted mean sensitivities and specificities for MRI and US were each more specific than sensitive, which contradicts recent meta-analyses which demonstrated sensitivities of 82.8% and 87.3% and specificities of 77.8% and 83.3% for MRI and US, respectively.^11,12^ This discrepancy may be due in part to our methods of data selection that prioritized higher specificity when multiple cutoffs/measurements were evaluated in a study. The ability of these modalities to identify structural anomalies and to provide visualization of the median nerve likely contributes to the lower false-positive rates we found with these modalities when selecting for higher specificity and thus utilizing more extreme diagnostic cutoffs. However, we still found that the sensitivity and specificity of MRI and US varied based on the reference standard used. Our data showed a decrease in specificity for both modalities when EDS is used as the reference standard. When clinical diagnosis was used as the reference standard, the specificity of MRI was nearly 100%, but specificity decreased to 87.6% when EDS was used as the reference standard. Although US noted a decrease in sensitivity when EDS was the reference standard, the sensitivity of MRI increased when EDS was the reference standard, although it remained relatively low at 77.1%. US has recently been proposed as an adjunct to EDS in CTS diagnosis given its occasional utility in identifying patients with false-negative EDS results,^6^ and this capability may have contributed to the decreased accuracy of the test when referenced against EDS as compared to clinical diagnosis. However, the 19.3%–9.1% false-negative rates of ultrasound and MRI demonstrated in our study favor the use of these tests as adjuncts when the clinical diagnosis/EMG is unclear or secondary causes are suspected, rather than as routine screening tests.

Beyond the presence of varying reference standards, comparison of CTS diagnostic modalities is inherently difficult due to varying diagnostic cutoff values and diverse methods of application for each diagnostic tool. In their evaluation of the utility of nerve conduction studies in CTS diagnosis, Demino et al. found similar difficulties in comparing the outcomes of different studies due to the wide variety of distal sensory latency and distal motor latency cutoff values used in various studies.^13^ Despite these drawbacks in systematically reviewing CTS diagnostic modality studies, our results identify some consistent characteristics within the diagnostic tools assessed. The sensitivity and specificity calculated for EDS was fairly similar to those identified by Nathan et al in their 1993 study on 2334 hands evaluated using clinical diagnosis as their reference standard (sensitivity 55.8%**–**86.3%, specificity 82.5%–94%).^14^ In addition, for cases of clinically clear-cut, severe CTS in which carpal tunnel release surgery is indicated, Jordan et al. found in their systematic review no statistically significant difference in surgical outcome between cohorts of patients with positive and negative electrodiagnostic tests.^15^ The congruence between EDS and clinical diagnosis combined with evidence suggesting that EDS is a poor predictor of surgical outcomes suggests that the widespread use of EDS to confirm a clinical diagnosis may incur extra expense, discomfort, and delay to treatment without adding valuable diagnostic support.

The current study had several limitations given the complexities of CTS diagnosis. Importantly, our decision to measure performance characteristics yielding the highest specificity may have underestimated the sensitivities of some tests. There was also significant heterogeneity in the criteria used to define clinical diagnosis, which limits the interstudy consistency among studies identified as using clinical diagnosis as a reference standard. Although nighttime or morning symptoms and a positive flick sign (paresthesia relieved by shaking the hand) have been cited as two of the most sensitive clinical aspects of CTS diagnosis, and thenar atrophy as one of the most specific, these were not consistently evaluated and uniformly utilized in the studies included in this review.^129–131^ Nonetheless, a constellation of signs and symptoms are required for CTS clinical diagnosis, which results in heterogeneity among the criteria used among the included studies. There was also variability in the metrics and cutoff values utilized for CTS diagnosis criteria for EDS, US, and MRI. Studies evaluating MRI used a range of variables including median nerve cross-sectional area, apparent diffusion coefficient, fractional anisotropy, and diffusion tensor coefficient. US was primarily evaluated using cross-sectional area of the median nerve or its percentage increase in area between the proximal and distal cross-sections; however, studies varied in measurement location and included the nerve at the level of the pisiform, the tunnel inlet, the distal outlet, etc. When evaluating EDS, radial-median latency, distal sensory latency, and/or median-ulnar latency were most used. Furthermore, few studies examining the same testing modality used identical metrics and cutoffs which further challenged comparisons from study to study. This variability limits the ability to generalize the diagnostic abilities of individual modalities and suggests that providers should cautiously interpret the cutoffs, criteria, and reference standards used in studies evaluating individual CTS diagnostic tools before adjusting their practice.

Overall, this study found that regardless of the reference standard used, MRI, US, and EDS all have false-positive and/or false-negative rates that discourage their use as screening modalities. Most testing characteristics, with the exception of MRI sensitivity, improved when clinical diagnosis was the reference standard. The lack of an agreed upon reference standard for CTS diagnosis makes comparison of testing performance characteristics challenging. However, this review demonstrates that regardless of the reference standard used, no diagnostic test performs well enough for use as a routine confirmatory test.

## DISCLOSURE

The authors have no financial interests to declare in relation to the content of this article.

## Supplementary Material


