# Supplementary material for: The Impact of Reference Standard on Diagnostic Testing Characteristics for Carpal Tunnel Syndrome: A Systematic Review
Source: Plast Reconstr Surg Glob Open. 2023 Jul 3;11(7):e5067. doi: 10.1097/GOX.0000000000005067 (PMC10317486; doi:10.1097/GOX.0000000000005067)
Supplement: Supplementary file 1 [file gox-11-e5067-s001.pdf]

SDC 1: Appendix. Clinical Diagnosis Definitions in Studies Using Clinical Diagnosis as the Reference Standard

Title	Year Published	Author(s)	# Patients Evaluated	# Wrists Evaluated	Diagnostic Tool Assessed	Clinical Diagnosis Definition
How to make electrodiagnosis of carpal tunnel syndrome with normal distal conductions?(17)	2010	Lee et al.	230	230	EDS	Presence of at least 1 of the following primary symptoms: (1) numbness, tingling pain, or paresthesia in the median nerve distribution; (2) precipitation of these symptoms by repetitive hand activities and relieved by resting, rubbing, and shaking the hand; (3) nocturnal awakening by such sensory symptoms.
The importance of ultrasound in diagnosis of carpal tunnel syndrome in Iraqi subjects (16)	2010	Bari et al.	150	265	EDS, US	Diagnosis based on clinical diagnostic criteria from American Academy of Neurology: paresthesia, pain, swelling, weakness, or clumsiness of the hand provoked or worsened by sleep, sustained hand or arm position, or repetitive action of the hand or wrist that is mitigated by changing posture or by shaking of the hand; sensory deficit or hypotrophy of the median innervated thenar muscle; symptoms elicited by the Phalen test, performed on each patient. Also performed the Tinel's & compression test.
Comparison of the diagnostic utility of Electromyography, Ultrasonography, Computed Tomography, Magnetic Resonance Imaging in idiopathic Carpal Tunnel Syndrome determined by clinical findings. (18)	2011	Deniz et al.	69	138	EDS, MRI, US	Weakness or atrophy of the opponens pollicis and/or the abductor pollicis brevis muscles or all of the following criteria together without muscle weakness: positive flick sign, median hypoesthesia, Tinel sign, Phalen sign, and reverse Phalen sign. CTS-negative wrists were wrists negative for all of the above
Color and power doppler US for diagnosing carpal tunnel syndrome and determining its severity: A quantitative image processing method (19)	2011	Ghasemi-Esfe et al.	156	156	US	Clinical evidence of CTS determined by an experienced neurologist and confirmed by an experienced hand surgeon, both university professors with more than 10 years clinical experience. All patients were selected from those with a classic presentation of CTS at a hand symptoms diagram test, and all patients had positive results at a Tinel or Phalen test, recurring activity-related or nighttime numbness, or tingling involving the palmar aspects of at least 2 radial fingers. The presence of median nerve sensory or motor deficit was also considered supportive of the diagnosis.

Combination of high-resolution and color Doppler ultrasound in diagnosis of carpal tunnel syndrome. (20)	2011	Ghasemi-Esfe et al.	134	134	US	Clinical evidence of CTS determined by an experienced neurologist and confirmed by an experienced hand surgeon, both university professors with more than 10 years clinical experience. All patients were selected from those with a classic presentation of CTS at a hand symptoms diagram test, and all patients had positive results at a Tinel or Phalen test, recurring activity-related or nighttime numbness, or tingling involving the palmar aspects of at least 2 radial fingers. The presence of median nerve sensory or motor deficit was also considered supportive of the diagnosis.
Sonographic and electrophysiological detection in patients with carpal tunnel syndrome (46)	2011	Guan et al.	60	114	EDS, US	at least one of the first two items and one of the rest five items below: (1) numbness in MN territory or the whole hand; (2) pain or hypesthesia in the hand; (3) awakened from sleep by numbness or pain at midnight or in the early morning; (4) numbness is relieved by shaking the hand, while aggravated by flexing the wrist, or more serious in winter than summer; (5) weakness of the hand; (6) atrophy of the thenar muscle; and (7) positive Phalen or Tinel sign
The segmental palmar test in diagnosing carpal tunnel syndrome reassessed (21)	2012	Kasius et al.	204	204	EDS	"Carpal tunnel syndrome was considered clinically present in patients with pain and/or paresthesias in and restricted to the sensory distribution of the median nerve (involvement of the fifth finger was an exclusion criterion), and if patients met 2 or more of the following criteria: (1) nocturnal paresthesias, (2) reproduction or aggravation of paresthesias or pain by provocative tests (Tinel or Phalen's sign), (3) aggravation of paresthesias by activities such as driving a car, riding a bike, holding a book, or holding a telephone, (4) paresthesias relieved by shaking the hand."
Comparing a new ultrasound approach with electrodiagnostic studies to confirm clinically defined carpal tunnel syndrome: a prospective, blinded study. (47)	2013	Claes et al.	210	264	US	Paresthesias or pain restricted to the median nerve innervated area with 2 or more of the following major criteria: (1) paresthesias or pain awakening the patient from sleep, (2) positive flick sign, and (3) aggravation by activities such as driving and holding a book or a telephone
Determination of the median nerve residual latency values in the diagnosis of carpal tunnel syndrome in comparison with other electrodiagnostic parameters (22)	2013	Khosrawi and Dehghan	119	164	EDS	Patients who had a positive history of pain or paresthesia in upper extremities and 2 of 3 signs suggesting CTS (Tinel's sign, median compression test, and Phalen's sign) were included as CTS group

Comparative study between physical examination, electroneuromyography and ultrasonography in diagnosing carpal tunnel syndrome (23)	2014	de Jesus Filho et al.	56	70	EDS, US	Complaints of pain or paresthesia on the path of the median nerve, with worsening at night, and presence of at least 1 positive clinical examination or evidence of atrophy in the thenar region
Validity of current electrodiagnostic techniques in the diagnosis of carpal tunnel syndrome (24)	2014	Eftekharsadat et al.	170	Not specified	EDS	Patients with at least 2 symptoms and/or one symptom and one sign of CTS considered as the case group; with the following: (1) Symptoms including hand numbness, tingling, paresthesia or nocturnal pain in the median nerve distribution area which were lasted for at least 3 months, (2) Signs including Tinel's sign or positive Phalen's test weakness especially abduction of digit 1 and the sensory deficit in the median territory
Comparison of ultrasound and electrodiagnostic testing for diagnosis of carpal tunnel syndrome: Study using a validated clinical tool as the reference standard (25)	2014	Fowler et al.	85	Not specified	EDS, US	CTS-6 score of ≥ 12
Comparison of peak versus onset latency measurements in electrodiagnostic tests for carpal tunnel syndrome(26)	2014	Kasius et al.	203	203	EDS	Paresthesias in the hand in a median nerve distribution, and at least 2 of the following major criteria: (1) paresthesias during night that awaken the patient from sleep, (2) paresthesias relieved by shaking the hand, and (3) aggravation of paresthesias by activities such as driving, bicycling, and holding a telephone book
What is the most sensitive test for diagnosing carpal tunnel syndrome? (27)	2014	Kodama et al.	137	168	EDS	2 or more of the following criteria: (1) paresthesias in the hand; (2) hypesthesias in the median distribution of the hand; (3) intermittent wrist and palm pain; (4) isolated weakness and atrophy of the abductor pollicis brevis muscle; (5) Tinel's or Phalen's signs
No correlation between sonographic and electrophysiological parameters in carpal tunnel syndrome. (45)	2014	Zyluk et al.	113	113	US	Diagnosis made on clinical grounds from the clinical history, symptoms, and signs.
Comparison of high-resolution sonography and electrophysiology in the diagnosis of carpal tunnel syndrome (28)	2015	Kanikannan et al.	107	192	EDS, US	Diagnosed by experienced neurologists based on clinical diagnosis criteria of (1) history of nocturnal or activity-related pain or dysesthesia limited to the hand, (2) sensory deficit in the median nerve distribution, (3) isolated weakness or atrophy of abductor pollicis brevis, and (4)

						positive Tine's or Phalen's sign. CTS were diagnosed when criteria 1 and one or more of criteria 2 to 4 were fulfilled.
Premotor potential study for diagnosis of carpal tunnel syndrome (29)	2016	Kodama et al.	168	168	EDS	≥2 of the following 5 criteria: (1) paresthesias of the hand, (2) hypesthesias in the median-nerve distribution of the hand, (3) intermittent wrist and palm pain, (4) isolated weakness and atrophy of the abductor pollicis brevis, and (5) positive Tinel's and/or Phalen's signs
The role of median nerve terminal latency index in the diagnosis of carpal tunnel syndrome in comparison with other electrodiagnostic parameters (30)	2016	Vahdatpour et al.	150	150	EDS	Positive history of paresthesia and paining upper extremities and 2 of 3 sign suggesting CTS ([1] Tinel's sign, [2] median compression test, [3] Phalen's test)
Role of diffusion tensor imaging in carpal tunnel syndrome: A case control comparative study to electrophysiological tests and clinical assessment. (42)	2018	Wafaie et al.	46	70	MRI	Clinically evident CTS as suggested by an expert orthopedic surgeon.The included candidates were presented with a typical clinical history,symptoms, and signs of CTS such as intermittent (nightly) numbness,tingling, or burning sensations in the thumb, index finger, middle finger, and the lateral half of the ring finger, atrophy of the thenar muscles and positive Phalen’s, Tinel’s and Durkan’s test results
A Prospective Comparison of Diagnostic Tools for the Diagnosis of Carpal Tunnel Syndrome (31)	2018	Wang et al.	250	408	EDS	Clinical diagnosis by a hand fellowship-trained orthopedic surgeon
Ultrasonography provides a diagnosis similar to that of nerve conduction studies for carpal tunnel syndrome (32)	2019	Drakopoulos et al.	192	192	EDS, US	wrist and hand pain, numbness, tingling, and/or burning in the median nerve distribution of the hand, a positive Phalen test, and a positive Tinel sign
Diagnostic potential of high resolution ultrasound and nerve conduction study in patients with idiopathic carpal tunnel syndrome (33)	2019	El-Shintenawy et al.	70	86	EDS, US	full history taking and clinical examination including motor and sensory examination and provocative tests as Tinnel’s sign, Phalen’s and direct compression tests
Comparative analysis of nerve conduction study methods in patients with carpal tunnel syndro me. (34)	2019	Jain et al.	120	182	EDS	Pain or paresthesia in hand (nocturnal or activity related) and at least 1 of the following: reduced 2-point discrimination or sensory impairment in the distribution of the median nerve, isolated atrophied abductor pollicis brevis muscle, Positive Tinel's or Phalen's sign

Motor Nerve Conduction Tests in Carpal Tunnel Syndrome (35)	2019	Kasius et al.	209	209	EDS	Pain and/or paresthesias in the sensory distribution of the median nerve, and 2 or more of the following (1) nocturnal paresthesias, (2) reproduction or aggravation of paresthesias by provocative tests (Tinel or Phalen signs); aggravation of pareesthesias by activities such as driving, riding a bike, holding a book or telephone; or (3) relief of symptoms by shaking the hand
The controversy of the normal values of ultrasonography in carpal tunnel syndrome: diagnostic accuracy of wrist-dependent CSA revisited (44)	2019	De Kleermaeker et al.	349	349	US	Pain or paresthesias in the territory innervated by the median nerve and if they met two or more of the following clinical CTS criteria: (1) nocturnal paresthesias, (2) aggravation of paresthesias by activities such as driving a car, riding a bike, holding a book, or holding a telephone, (3) paresthesias relieved by shaking the hand (the positive flick sign)
Emerging role of ultrasonography in the diagnosis of carpal tunnel syndrome: Relation to risk factors, clinical and electrodiagnostic severity (37)	2020	El-Najjar et al.	60	60	EDS, US	History and physical examination in accordance with criteria proposed by Keith et al. in *Diagnosis of carpal tunnel syndrome.*
The prognostic value of median nerve thickness in diagnosing carpal tunnel syndrome using magnetic resonance imaging: a pilot study (43)	2020	Lee et al.	40	40	MRI	Positive CTS sign (Tinel's sign, Phalen's test or modified Phalen's test)
Augmented Diagnostic Ac curacy of Ultrasonography for Diagnosing Carpal Tunnel Syndrome Using an Optimised Wrist Circumference-Dependent Cross-Sectional Area Equation (49)	2020	Olde-Dubbelink et al.	349	349	US	Pain and/or paresthesia in the territory innervated by the median nerve, and 2 or more of the following: (1) nocturnal paresthesia, (2) aggravation of paresthesia by activities such as driving a car, riding a bike, holding a book, or holding a telephone, (3) positive flick sign.
Value of ultrasonography in the diagnosis of carpal tunnel syndrome-a new ultrasonographic index in carpal tunnel syndrome diagnosis: A clinical study (50)	2020	Pertea et al.	344	395	US	Clinical features of pain and paresthesia more intense during mornings, sensory symptoms in the median nerve territory, decreased muscle strength. Provocative tests (Tinel, Phalen,Durkan) as well as those to discriminate tactile, painful and thermal sensitivity were used. The two-point tactile sensitivity discrimination test (2PD) and Simmens-Weinstein (SW) monofilament tests were also used. The

						color and humidity of hand and finger skin were evaluated, as well as the presence or absence of hand muscle atrophy. History taking included the detection of professional factors, activity characterized by frequent repetitive movements and traumatic antecedents.
Role of High Frequency Ultrasound in Diagnosing Carpal Tunnel Syndrome as Compared with Conventional Nerve Conduction Studies (39)	2020	Singla et al.	56	106	EDS, US	Diagnosis based on the American Academy of Neurology clinical diagnostic criteria: paresthesia, pain, swelling, weakness or clumsiness of the hand provoked or worsened by sleep; sustained hand or arm position; repetitive action of the hand or wrist that is mitigated by changing posture or by shaking of the hand; sensory deficit or hypotrophy of the median innervated thenar muscle; symptoms elicited by Phalen test
Application of the calculated electrophysiological parameters in early diagnosis of carpal tunnel syndrome (37)	2020	Zhang et al.	75	104	EDS	Diagnosis based on clinical diagnostic criteria from American Academy of Neurology: (1) paresthesia, pain, swelling, weakness, or clumsiness of the hand provoked or worsened by sleep, sustained hand or arm position, or repetitive action of the hand or wrist mitigated by a change in posture or by shaking of the hand; (2) sensory deficits in the median nerve innervated regions of the hand; (3) motor deficit or hypotrophy of the median nerve innervated muscles; and (4) positive provocative clinical tests (positive Phalen and/or Tinel sign). Subjects diagnosed CTS if they met item (1) together with one or more items from (2) to (4).
Accuracy of high-resolution ultrasonography in establishing the diagnosis of carpal tunnel syndrome (36)	2020	Aggarwal et al.	100	Not specified	EDS, US	Diagnosis made clinically according to the diagnostic criteria of 'American Academy of Neurology' (including symptoms, provocative factors, mitigating factors, and physical examination)
Value of Grayscale and Power Doppler High-Resolution Ultrasound in Assessment of Patients with Clinically Suspected Carpal Tunnel Syndrome (38)	2020	Gamil et al.	91	144	EDS, US	Cases diagnosed based on clinical grounds, as indicated by history taking (nocturnal discomfort and sensory impairment in the MN distribution with or without motor impairment) and a physical examination (including provocative Phalen and Tinel Tests)

Posterior Border Distance: An Effective Diagnostic Measurement for Carpal Tunnel Syndrome Using Ultrasonography (48)	2020	Meric et al.	53	Not specified	US	CTS diagnosed based on 2 or more of the following criteria: i) increased paresthesia confirmed by provocative tests (Phalen's/Tinel's signs), i) nocturnal paresthesias, iii) increased pain and numbness while holding a book or phone, cycling, and driving, and iv) regression of symptoms by shaking hands
Prolonged median distal sensory nerve action potential duration in carpal tunnel syndrome (41)	2021	Yang et al.	324	324	EDS	(1)Paresthesia/dysesthesia of the hand that can be relieved by vigorous shaking of the hand, worse at night and aggravated by heavy manual or repetitive hand and wrist actions and one or more of the following criteria: (2) intermittent tingling, pain, or altered sensation of the fingers in the distribution of the median nerve; (3) thenar muscle weakness or atrophy and reduced hand dexterity; and (4) abnormal results in 2 or more of the following: Tinel's sign, modified Phalen's and Durkan's compression tests.
